# Microcirculation-driven mitochondrion dysfunction during the progression of experimental sepsis

**DOI:** 10.1038/s41598-024-57855-9

**Published:** 2024-03-26

**Authors:** Roland Fejes, Attila Rutai, László Juhász, Marietta Zita Poles, Andrea Szabó, József Kaszaki, Mihály Boros, Szabolcs Péter Tallósy

**Affiliations:** Institute of Surgical Research, Albert Szent-Györgyi Medical School, University of Szeged, Szeged, 6720 Hungary

**Keywords:** Mitochondria, Bacterial infection, Preclinical research, Blood flow

## Abstract

Sepsis is accompanied by a less-known mismatch between hemodynamics and mitochondrial respiration. We aimed to characterize the relationship and time dependency of microcirculatory and mitochondrial functions in a rodent model of intraabdominal sepsis. Fecal peritonitis was induced in rats, and multi-organ failure (MOF) was evaluated 12, 16, 20, 24 or 28 h later (n = 8/group, each) using rat-specific organ failure assessment (ROFA) scores. Ileal microcirculation (proportion of perfused microvessels (PPV), microvascular flow index (MFI) and heterogeneity index (HI)) was monitored by intravital video microscopy, and mitochondrial respiration (OxPhos) and outer membrane (mtOM) damage were measured with high-resolution respirometry. MOF progression was evidenced by increased ROFA scores; microcirculatory parameters followed a parallel time course from the 16th to 28th h. Mitochondrial dysfunction commenced with a 4-h time lag with signs of mtOM damage, which correlated significantly with PPV, while no correlation was found between HI and OxPhos. High diagnostic value was demonstrated for PPV, mtOM damage and lactate levels for predicting MOF. Our findings indicate insufficient splanchnic microcirculation to be a possible predictor for MOF that develops before the start of mitochondrial dysfunction. The adequate subcellular compensatory capacity suggests the presence of mitochondrial subpopulations with differing sensitivity to septic insults.

## Introduction

The progression of sepsis is accompanied by an imbalance between oxygen delivery (DO_2_) and consumption (VO_2_), and the associated microcirculatory-mitochondrial dysfunction syndrome (MMDS) is generally considered to be the driver of multi-organ failure (MOF)^1,2^. The advancing feature of this process implies ongoing changes or amplifying loops which may involve the main MMDS components towards organ malfunction to different degrees^3–7^. Nevertheless, the onset of MOF is a definite turning point in the trajectory of events, since “before and after MOF” periods have different impacts on the outcome^8^. Certainly, this issue is of critical importance as clinical diagnostics and therapeutic decisions can all be tailored or fine-tuned to the complementary parts of a progressive syndrome.

It should be noted here that systemic hemodynamics and global oxygenation parameters usually do not follow the progression tightly, and microcirculatory oxygen transport in this scheme seems relatively independent of macrohemodynamic variables^9,10^. Similarly, it has been proposed that microcirculatory status is a better predictor not only of the severity of MOF but also of sepsis mortality. Indeed, algorithms that support microcirculatory DO_2_ for an upkept mitochondrial VO_2_ are in the focus of clinical and basic research interest^11^.

It should also be added that increased cardiac output and higher metabolic rate with preserved capillary perfusion in vital organs usually prevail during the acute hyperdynamic phase of sepsis^12–15^. The early mitochondrial reaction is largely tissue-dependent, but in most cases, it is characterized by increased VO_2_ and a shift to a catabolic state. Thereafter, a plateau is reached and the hypodynamic phase involves reduced cardiac performance, tissue hypoperfusion with hyperlacticemia and other signs of mitochondrial and organ damage^16,17^. In this simplified outline, tissue mitochondria together with their supplier microcirculatory network are both significant driving forces for the development of MOF. However, it remains an open question whether these changes are causally related and, if so, how this bidirectional connection develops in time. Indeed, there are three possibilities for mitochondrial dysfunction: it may be a direct and early consequence of microcirculatory failure, thus being the promoter of progression; it can occur in parallel with microcirculatory events, interacting continuously in a vicious circle and influencing the outcome jointly; or, theoretically, it can occur independently of microcirculatory impairment.

It is challenging to demonstrate causal links between the microcirculatory and mitochondrial arms of MMDS even in standardized experimental conditions due to the web of connections and cross-reactions which make it difficult to interpret findings accurately. Of note, the microcirculatory and mitochondrial changes have not been investigated simultaneously in human sepsis in the pre- and post-MOF periods^18–20^. We have therefore designed an in vivo study to monitor the splanchnic microvascular status and the concurrent mitochondrial function simultaneously, together with other signs of the septic response in an accepted preclinical model of intraabdominal sepsis. In this longitudinal protocol the animals were randomly allocated into independent groups during different periods (from 12 to 28 h) of sepsis progression. This protocol allowed us to investigate the correlation between established variables of microcirculatory or mitochondrial dysfunction and to determine their predictive diagnostic value in the course of experimental sepsis.

## Results

### Animal well-being and time-dependent changes in multi-organ failure markers

**Figure 1:**
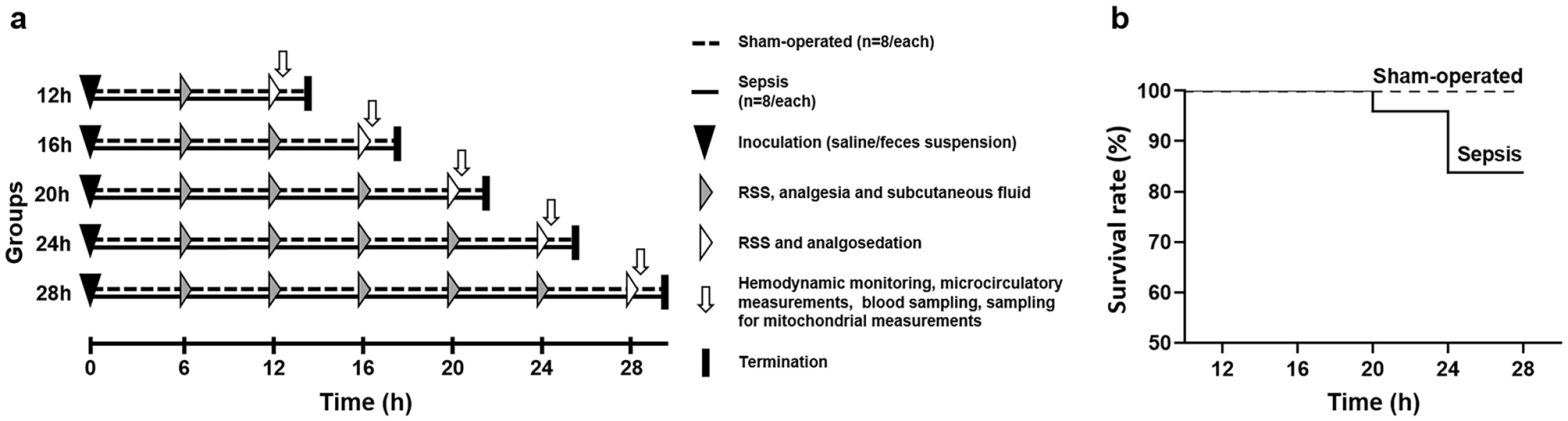
The experimental protocol and mortality during the study. The experimental protocol (**a**) shows groups, interventions and assessments. The animals were randomly assigned to sham-operated and septic groups, which were further divided into five subgroups each according to the length of sepsis progression (12th, 16th, 20th, 24th and 28th h). Interventions and assessments are marked with symbols. Kaplan–Meier analysis (**b**) was performed to describe survival of experimental animals. One animal in the 20th h septic and two animals in the 24th h septic group were sacrificed due to the critical level of suffering caused by sepsis induction.

Assessments of animal well-being and organ dysfunction were performed using RSS scores (rat-specific, well-being-related sickness score) and the ROFA scoring (rat-specific organ failure assessment) system, respectively, while the termination of experiments was scheduled at predetermined time points (12th h, 16th h, 20th h, 24th h and 28th h) after sepsis induction (Fig. 1a). There were no significant changes in the RSS score in the sham-operated group. Septic groups between the 20th and 24th h elevated significantly not only compared to the 12th h septic sham-operated group but also compared to the 20th and 24th h sham-operated groups (see Supplementary Fig. S1 online). Three animals were euthanized (one at the 20th h and two more at the 24th h) due to the critically elevated RSS score (at least 6 points). The number of euthanized animals is included in the mortality calculations (Fig. 1b). IL-6 significantly increased as compared to the control group, peaking at the 16th h (P < 0.001), after which a decrease started reaching the level of the sham-operated group after the 24th h (Table 1). ROFA parameters were significantly higher in the septic groups compared to the sham-operated animals during the whole experimental period, with the score reaching its maximum at the 20th h (P = 0.007; Table 1).

**Table 1:** Inflammatory biomarkers, parameters of oxygen dynamics, ROFA scores and components in sham-operated and sepsis groups with different observation periods.

Parameters	Groups	Time (h)				
		12	16	20	24	28
Cardiac output (mL/min/kg)	Sham-operated	132.00 (119.75; 140.50)	124.00 (112.25; 132.50)	125.00 (118.00; 134.25)	121.50 (116.00; 131.50)	129.50 (113.75; 138.25)
	Septic	136.50 (133.25; 151.75)	155.00 (148.25; 171.00)*^x^	96.00 (87.00; 108.00)*^x^	**120.50 (117.00; 126.50)**^**x**^	131.50 (116.00; 139.50)
Mean arterial pressure (mm Hg)	Sham-operated	110.00 (99.75; 123.00)	105.50 (102.00; 125.75)	108.00 (99.75; 119.25)	108.00 (103.50; 115.75)	101.00 (97.50; 105.50)
	Septic	100.50 (93.75; 104.50)	100.50 (89.50; 105.00)*	82.00 (70.00; 92.00)*^x^	88.00 (69.25; 97.25)*	112.50 (97.75; 133.25)
Oxygen extraction (%)	Sham-operated	21.77 (18.31; 33.52)	12.12 (9.47; 19.71)	30.89 (23.34; 41.56)	17.77 (11.35; 25.53)	15.54 (12.03; 19.98)
	Septic	8.62 (7.22; 11.51)*	33.31 (13.88; 45.02)*^x^	**37.01 (15.21; 50.52)**^**x**^	**23.66 (14.66; 47.39)**^**x**^	13.72 (11.14; 31.61)
Oxygen delivery (mL/min/1,000)	Sham-operated	66.48 (57.87; 81.11)	63.31 (52.45; 79.06)	64.82 (57.39; 71.51)	63.93 (55.86; 70.82)	65.10 (59.13; 75.59)
	Septic	105.12 (91.22; 110.67)*	**80.21 (65.65; 85.27)**^**x**^	46.135 (37.54; 55.02)*^x^	**54.40 (37.95; 61.37)**^**x**^	88.57 (73.17; 98.35)*
Oxygen consumption (mL/min/1,000)	Sham-operated	13.97 (12.86; 22.52)	7.56 (5.67; 18.65)	20.13 (16.04; 27.75)	10.15 (8.42; 17.16)	9.85 (7.84; 12.15)
	Septic	9.34 (6.84; 15.64)	27.22 (11.36; 29.54)*^x^	16.42 (6.34; 28.68)	12.80 (6.254; 22.523)	12.15 (10.89; 23.80)
Plasma IL-6 (pg/mL)	Sham-operated	8.2 (1.64; 13.74)	3.18 (0.72; 12.25)	8.62 (1.76; 17.65)	18.85 (10.03; 32.25)	3.18 (0.650; 9.09)
	Septic	18.45 (13.85; 51.03)*	52.25 (13.53; 95.33)*	49.00 (18.70; 68.00)*	16.05 (11.70; 39.88)	40.85 (5.68; 62.60)*
Whole blood lactate (mmol/L)	Sham-operated	0.90 (0.90; 0.90)	0.00 (0.00; 0.00)	0.90 (0.90; 0.90)	1.20 (0.00; 1.58)	0.85 (0.45; 1.33)
	Septic	1.55 (1.38; 1.95)	3.25 (2.14; 3.83)*	3.60 (2.25; 4.65)	2.75 (2.48; 2.95)	1.60 (0.80; 2.25)
PaO_2_/FiO_2_ ratio	Sham-operated	406.95 (402.45; 420.88)	401.00 (383.10; 402.45)	355.71 (338.93; 369.41)	395.62 (357.98; 401.25)	373.83 (332.26; 405.81)
	Septic	363.14 (332.14; 383.45)	292.33 (277.62; 327.80)*	348.00 (305.17; 374.76)	291.19 (221.99; 311.07)	364.05 (322.26; 375.24)
Plasma ALT (U/L)	Sham-operated	33.00 (30.13; 35.25)	44.00 (39.00; 47.75)	41.50 (35.75; 45.50)	41.00 (31.00; 51.00)	37.00 (46.75; 42.25)
	Septic	40.50 (36.50; 49.50)	35.50 (33.00; 70.50)	**24.00 (22.25; 25.00)**^**x**^	31.00 (26.00; 32.50)	51.50 (42.25; 60.75)
Plasma AST (U/L)	Sham-operated	44.17 (40.74; 52.15)	65.52 (59.19; 85.05)	71.57 (54.74; 93.06)	73.80 (54.77; 90.95)	48.77 (37.49; 79.94)
	Septic	92.55 (71.43; 118.73)*	145.20 (91.65; 176. 91)	44.69 (36.55; 72.44)	50.40 (42.30; 78.09)	90.14 (60.88; 115.41)
Plasma urea (mmol/L)	Sham-operated	5.55 (4.38; 6.85)	4.95 (4.55; 6.55)	5.10 (4.38; 5.88)	4.90 (4.23; 6.90)	6.45 (5.10; 7.28)
	Septic	9.55 (5.60; 11.83)	7.75 (5.85; 10.90)	7.60 (6.30; 20.10)	8.25 (6.63; 9.20)	6.30 (5.43; 8.70)
ROFA score	Sham-operated	0.50 (0.00; 1.00)	1.00 (0.00; 1.00)	1.00 (0.25; 2.00)	1.00 (1.00; 2.00)	1.00 (0.25; 1.38)
	Septic	3.00 (2.25; 3.75)*	6.00 (5.00; 7.00)*^x^	7.00 (5.00; 8.00)*^x^	5.50 (4.75; 7.25)*^x^	2.50 (2.00; 3.00)*

Data demonstrate the median and 25th and 75th percentiles. Comparison between groups was conducted with the Kruskal–Wallis test followed by Dunn’s post-hoc test. *P < 0.05 vs sham-operated groups (in boldface); ^x^*P* < 0.05 vs 12 h sepsis (between sepsis groups) (in boldface).

### Time-dependent changes in macrohemodynamics

The changes in macrohemodynamic parameters are demonstrated in Table 1. In the sham-operated group, there were no significant hemodynamic changes over time. Sepsis was characterized by significant hypotension between the 16th and 24th h (P_16h_ = 0.05; P_20h_ < 0.001; P_24h_ = 0.001) compared to the sham-operated animals in the same time points. An increasing trend was observed in CO data until the 16th h (P < 0.001), and then CO dropped significantly below the level of the sham-operated animals at the 20th h (P < 0.001). Later, CO values did not differ from the controls. In the 16–24 h interval, the CO values differed significantly from the values of the 12th h group, albeit in a different way (P_16h_ = 0.009; P_20h_ < 0.001; P_24h_ = 0.008).

### Time-dependent changes in oxygen dynamics

The numerical parameters of oxygen dynamics are indicated in Table 1. DO_2_ showed a progressive decline in the septic group, reaching its lowest value at the 20th h, thereafter exceeding the levels for sham-operated animals at the 28th h. There was no significant change in VO_2_ except at the 16th h (P = 0.008). ExO_2_ rose until the 20th h when the trend reversed and did not alter from the values of the sham-operated groups. In the 16th–24th h interval, the ExO_2_ values differed significantly from the values of the 12th h septic group (P_16h_ = 0.035; P_20h_ < 0.005; P_24h_ = 0.039).

### Time-dependent changes in microhemodynamics

**Figure 2:**
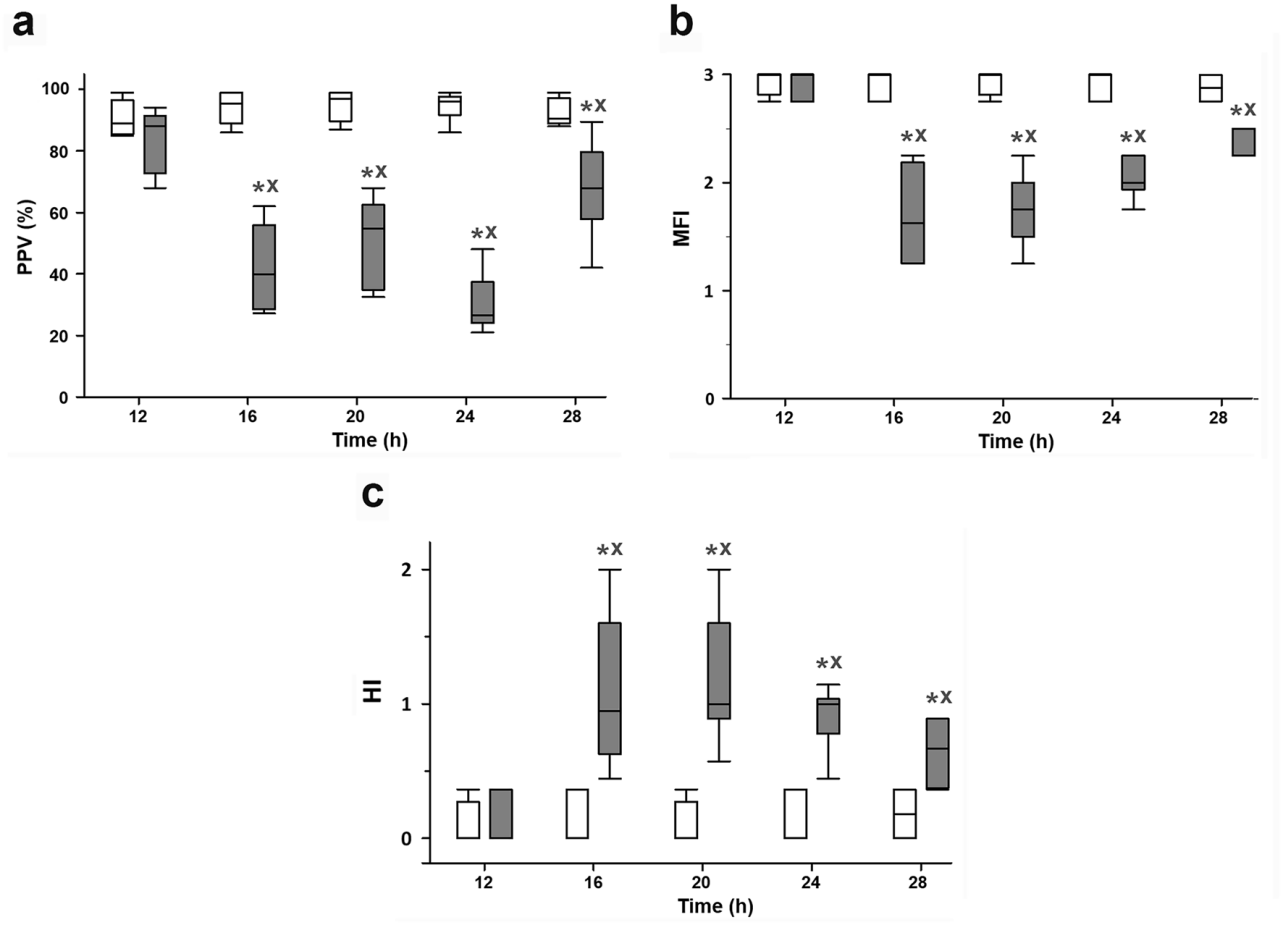
Proportion of perfused vessels (PPV;** a**), microvacular flow index (MFI; **b**) and heterogeneity index (HI; **c**) in the sham-operated animal subgroups (white boxes) and in the sepsis subgroups (grey boxes). The plots demonstrate the median (horizontal line in the box) and the 25th (lower whisker) and 75th (upper whisker) percentiles. Data were analyzed by two-way analysis of variance (ANOVA) followed by the Holm–Sidak post-hoc test. *P < 0.05 vs. sham-operated at the same time point, ^X^*P* < 0.05 vs. the 12th h sham-operated group.

PPV (P_16h–28h_ < 0.001; Fig. 2a) and MFI were significantly higher (P_16h–28h_ < 0.001; Fig. 2b) as compared with those in the sham-operated group between the 16 and 28 h interval. HI was significantly higher in the 16–28 h time window with a decreasing trend in the septic group, but still being significantly higher compared to the sham-operated group at the 28th h (P_28h_ = 0.003; Fig. 2c).

### Time-dependent changes in liver mitochondrial functions

**Figure 3:**
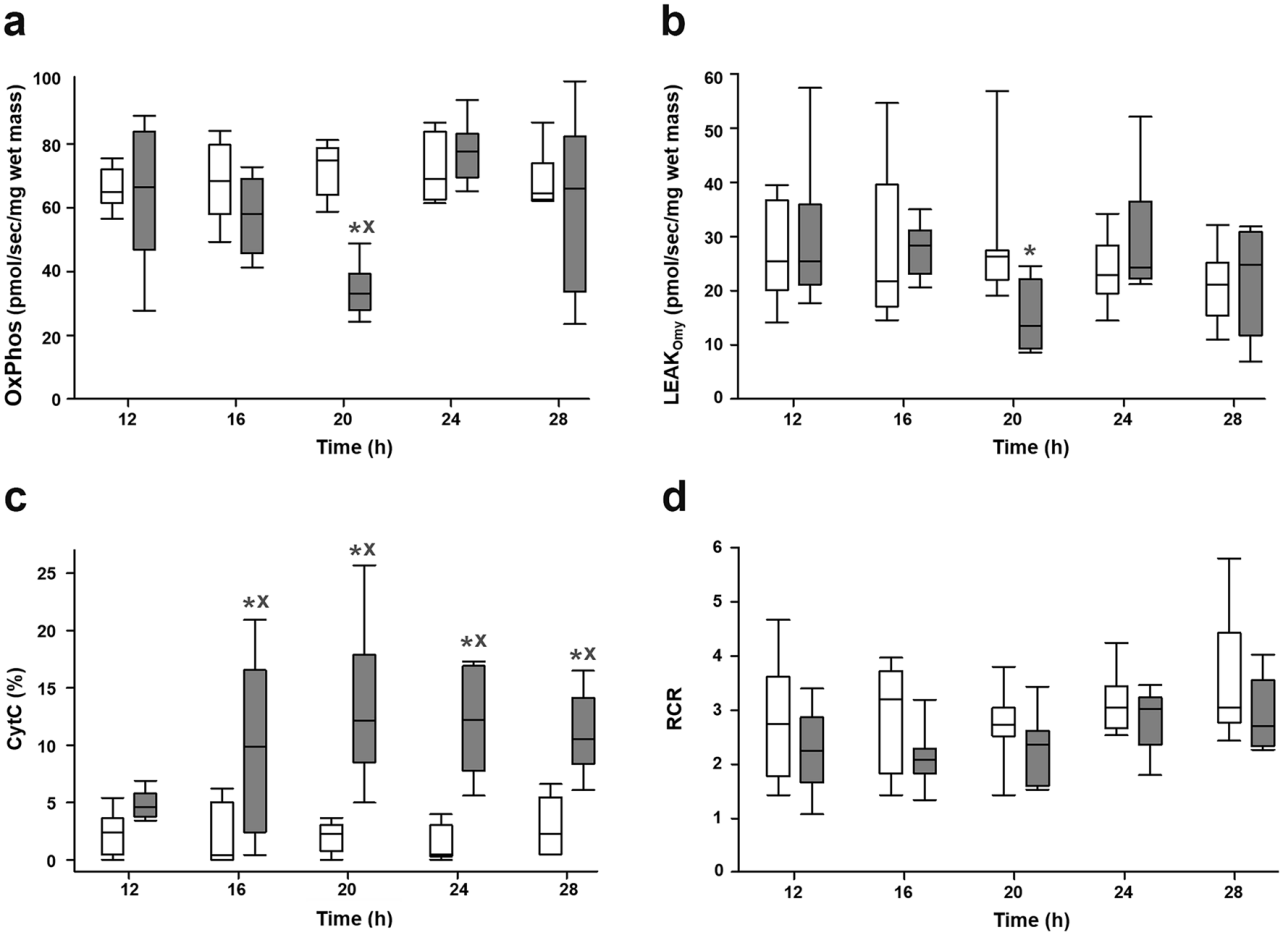
Oxidative phosphorylation (OxPhos;** a**), oligomycin-induced leak state (LEAK_Omy_;** b**), cytochrome c control efficiency (CytC%;** c**) and respiratory control ratio (RCR;** d**) in the sham-operated animal subgroups (white boxes) and in the sepsis subgroups (grey boxes). The plots demonstrate the median (horizontal line in the box) and the 25th (lower whisker) and 75th (upper whisker) percentiles. Data were analyzed by two-way analysis of variance (ANOVA) followed by the Holm–Sidak post-hoc test. *P < 0.05 vs. sham-operated at the same time point, ^X^*P* < 0.05 vs. the 12th h sham-operated group.

OxPhos and LEAK_Omy_ values of septic animals significantly decreased at the 20th h (OxPhos: P < 0.001; LEAK_Omy_: P = 0.006) compared to the sham-operated group (Fig. 3a,b). CytC%, which characterizes mtOM damage, showed a strong increasing trend with a maximum at the 20th h (P = 0.028) and was significantly different from the sham-operated groups between the 16th and the 28th h (P_16h–28h_ < 0.001; Fig. 3c). RCR did not differ significantly in the septic animals compared to the sham-operated ones during the whole experimental period (Fig. 3d).

### Relative changes in functional parameters over time

The changes in circulatory and mitochondrial parameters as a function of time were examined together with ROFA scores, and significant differences were observed as compared to the sham-operated group. The most marked changes in microcirculatory parameters were observed in the heterogeneity index as an eightfold change between the 16th and 20th h period. Compared to the baseline (12th h), the parameters of the other time points showed the least change in macrocirculation, while more pronounced changes were observed in the ROFA score and mitochondrial parameters (see Supplementary Fig. S2 online).

### Receiver operating characteristic (ROC) curve analyses

**Figure 4:**
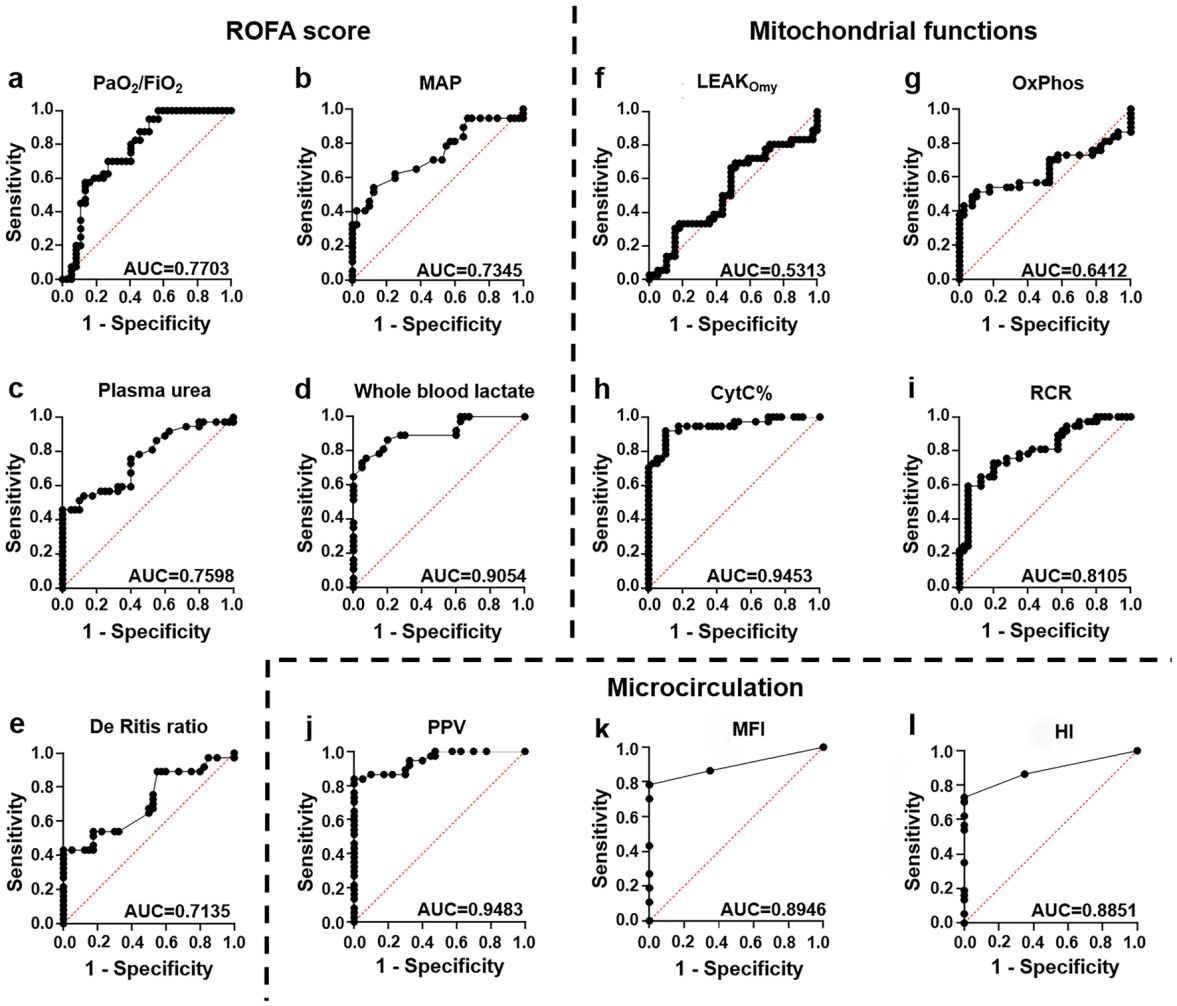
ROC curves provide information about the predictive diagnostic strength of the given parameters. ROC curve of PaO_2_/FiO_2_ ratio (**a**), mean arterial pressure (MAP; **b**), plasma urea level (**c**), whole blood lactate level (**d**), DeRitis ratio (**e**), oligomycin-induced leak respiration (LEAK_Omy_; **f**), oxidative phosphorylation (OxPhos; **g**), cytochrome c control efficiency (CytC%; **h**), respiratory control ratio (RCR; **i**), proportion of perfused vessels (PPV; **j**), microvascular flow index (MFI; **k**) and heterogeneity index (HI; **l**).

The diagnostic power of the parameters was determined with the calculated AUC values from the ROC analysis. Among the parameters of ROFA score (Fig. 4a–e), only whole blood lactate level showed relevant predictive value (AUC_lactate_ = 0.9054). Among the mitochondrial functions (Fig. 4f–i) CytC% proved to be a strong predictor (AUC_CytC%_ = 0.9453). Among microcirculatory parameters (Fig. 4j–l), PPV showed the strongest diagnostic value (AUC_PPV_ = 0.9483), however both MFI and HI had a particularly high diagnostic value (AUC_MFI_ = 0.8946; AUC_HI_ = 0.8851).

### Correlation between mitochondrial and microcirculatory parameters and the ROFA score

**Figure 5:**
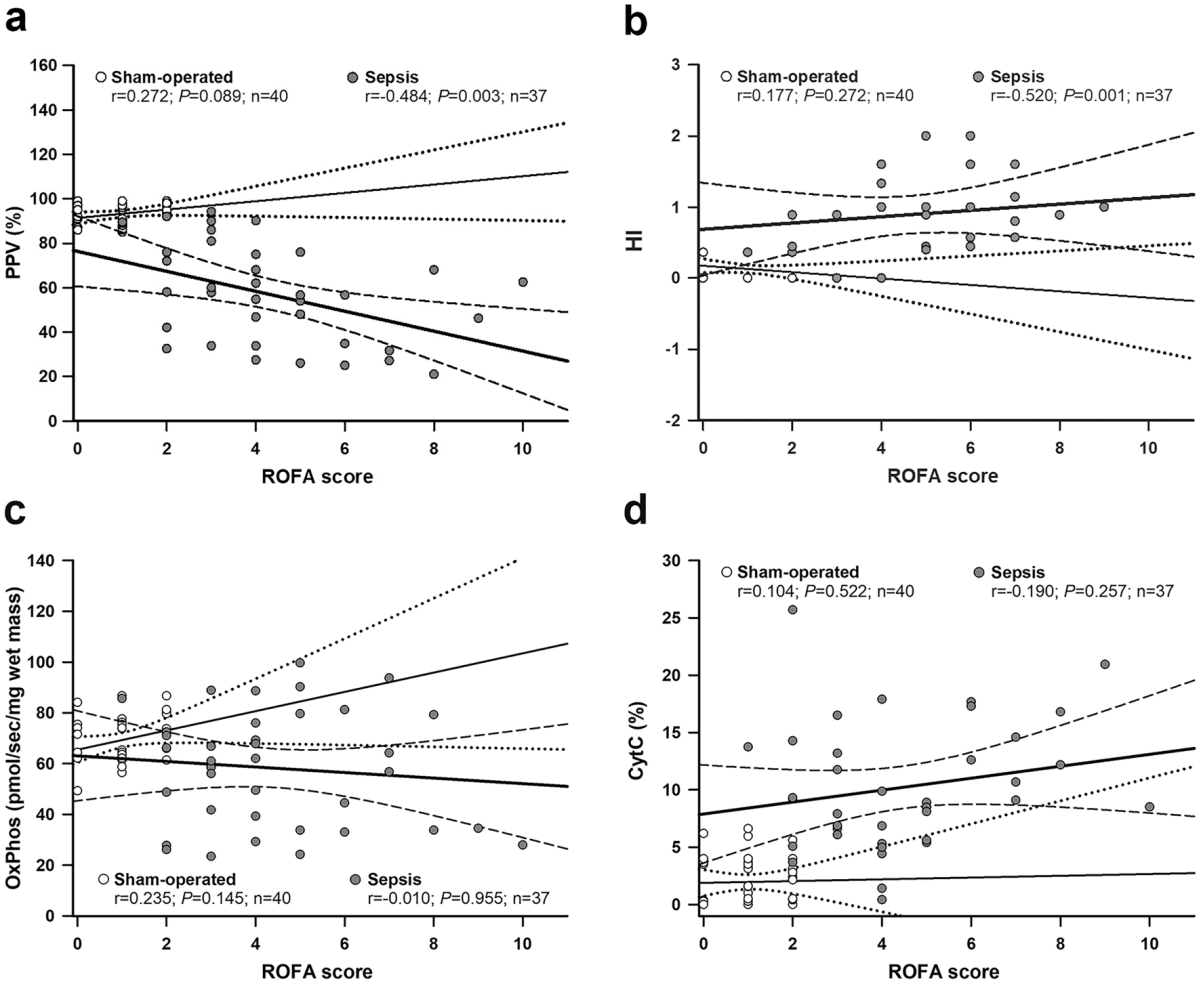
Correlation between the ROFA score and microcirculatory parameters (**a:** proportion of perfused vessels [PPV]; **b:** heterogeneity index [HI]) and mitochondrial functions (**c:** oxidative phosphorylation [OxPhos]; **d:** cytochrome c control efficiency [CytC%]) were examined by the Spearman’s correlation test. Correlation coefficient r values, (null hypothesis-related) P values and numbers of animals involved in the sham-operated and septic groups are provided with a 95% confidence interval. The sham-operated subgroup (n = 40) is marked with open circles, a thin regression line and a dotted line for the 95% confidence interval, whereas the septic subgroup (n = 37) is indicated with dark grey circles, a thick regression line and a dashed line for the 95% confidence interval.

There was no significant correlation in the sham-operated groups. There was a negative, significant fair correlation between PPV and the ROFA score (r = − 0.484, P = 0.00259; Fig. 5a) and a positive, significant moderate correlation between HI and the ROFA score (r = 0.520, P = 0.00106; Fig. 5b). There were no relations between the mitochondrial parameters and the ROFA score (Fig. 5c,d).

### Correlation between mitochondrial and microcirculatory parameters

**Figure 6:**
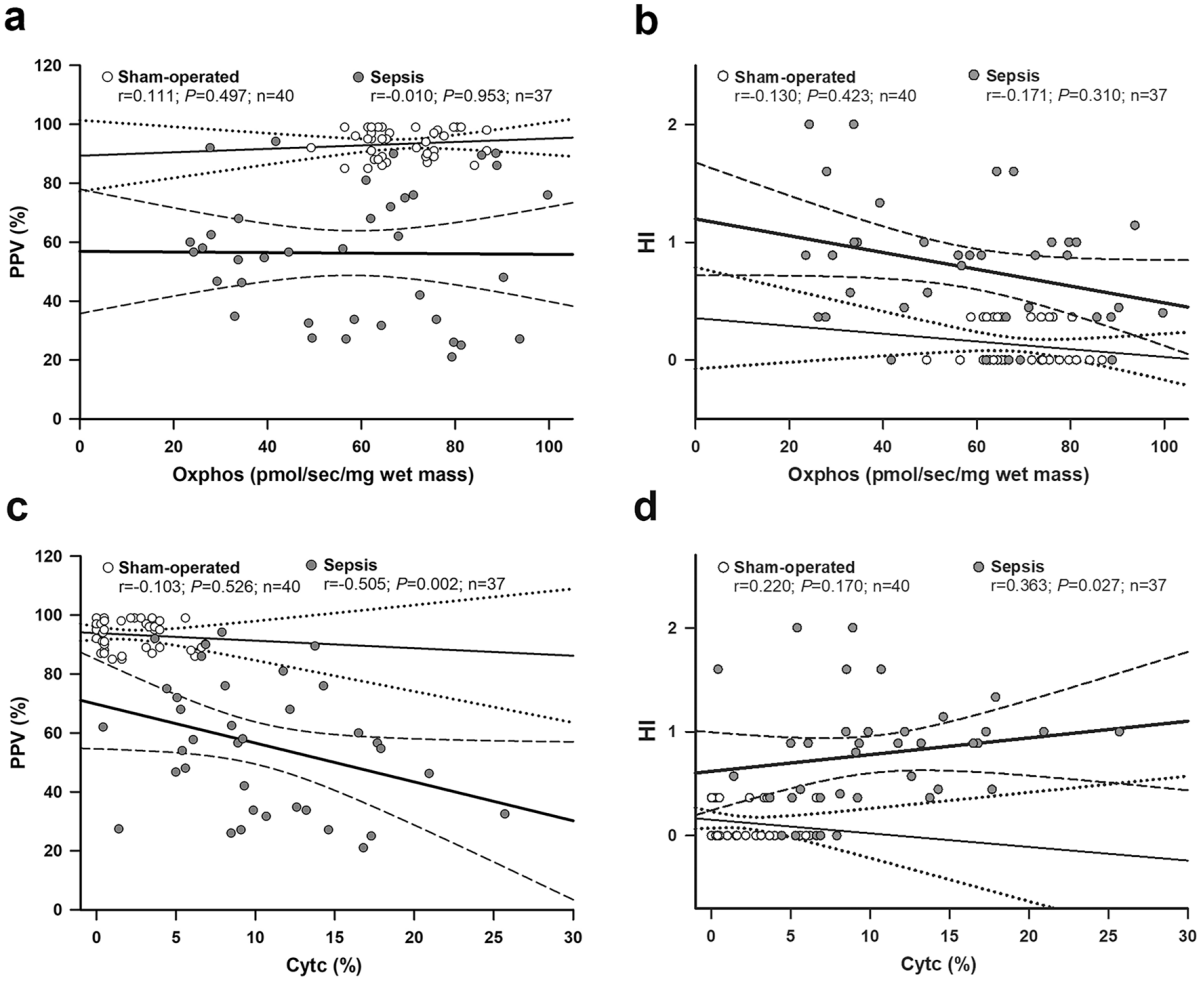
Correlation between oxidative phosphorylation (OxPhos) and proportion of perfused vessels (PPV; **a**) and heterogeneity index (HI;** b**) and between cytochrome c control efficiency (CytC%) and PPV (**c**) and HI (**d**) were examined by the Pearson correlation test. Correlation coefficient r values, (null hypothesis-related) P values and numbers of animals involved in the sham-operated and septic groups are provided with a 95% confidence interval. The sham-operated subgroup (n = 40) is marked with open circles, a thin regression line and a dotted line for the 95% confidence interval, whereas the septic subgroup (n = 37) is indicated with dark grey circles, a thick regression line and a dashed line for the 95% confidence interval.

There was no significant correlation in the sham-operated groups. There was no significant correlation between the microcirculation and OxPhos variables (Fig. 6a,b). There was a negative, significant moderate correlation between PPV and CytC% (r = − 0.505, P = 0.00153; Fig. 6c); however, HI and CytC% also showed a moderate and positive relationship (r = 0.332, P = 0.0445; Fig. 6d).

## Discussion

This research protocol was designed to define a possible relationship between microcirculatory and mitochondrial components of the pathobiology of evolving septic reaction as a function of time. The study design strictly adhered to the latest experimental recommendations, and the ensuing septic response followed the expected patterns to reproduce a moderately severe condition. The rodent fecal peritonitis model meets reproducibility and standardization criteria and offers close similarity to a realistic clinical scenario with no source control^8,9,21,22^.

Our study presents a hypodynamic model of septic shock since CO declined below the level of the sham-operated group while the increase in CO was observed only at the 16th h. Remarkably, the impaired intestinal microcirculation persists from the start of the study, despite the simultaneous increase in ExO_2_, which may indicate sustained cellular metabolic activity. Compared with other findings in the literature, our results are consistent with increased capillary O_2_ extraction in early sepsis and emphasize microcirculatory dysfunction as a central component of compromised DO_2_^23^. Recent studies also confirm microvascular dysfunction preceding detectable tissue damage^24^.

In this setup, the divergent dynamics of microcirculatory and mitochondrial responses were clearly discerned with the presence of the whole septic macrohemodynamic pattern with compensatory hyperdynamic, decompensated hypodynamic and final recovery stages which provide insight into the progression of sepsis without resuscitation. The joint feature of microcirculatory-mitochondrial impairment, or MMDS, has already been identified as a significant element in the pathogenesis of human MOF, but alterations to intestinal microcirculation and hepatic mitochondrial functions to date have not been studied together in MOF associated with intraabdominal sepsis^22,25,26^. In theory, a septic insult can affect all of the components of a microcirculatory system, and an increased capillary leakage, glycocalyx damage or intravascular abnormalities can all lead to reduced ExO_2_ by the decreased oxygen diffusion and DO_2_ within the affected tissues^23,27–30^. Our study partially confirms this notion, showing an early proportionate decrease of perfused microvessels and increased perfusion heterogeneity with matching changes in ROFA scores. Nevertheless, the initial microcirculatory failure was improved and then restored in the later phase of the 28 h observation period. Importantly, the hepatic mitochondrial function deteriorated much later with a rapid recovery of oxidation-linked parameters and a permanently sufficient coupling of the respiratory chain, but CytC% was elevated significantly, implying mtOM damage. CytC is an electron shuttle protein loosely anchored to the mitochondrial inner membrane. Injury to mtOM definitely leads to the partial loss of intramitochondrial CytC and decreased mitochondrial VO_2_^31–33^. We have used an established respirometry method to determine mtOM integrity with stimulation of mitochondrial respiration following CytC replacement from an outer source^34,35^. In theory, damage to the mtOM should also involve impairment of mitochondrial coupling, but this was not the case here. CytC% had a high diagnostic power during the whole course of the 28 h experimental period, thus suggesting that it may be a suitable biomarker for the onset of sepsis. However, despite this correlation, CytC% or OxPhos changes did not influence the ROFA score. Our results are consistent with the literature in terms of septic mtOM damage and deterioration of oxidative functions of the respiratory chain. However, the markedly rapid restoration of OxPhos suggests an immense reserve capacity of hepatic mitochondrial respiration to compensate for the loss of membrane integrity. Of note, the correlation between ROFA scores and microcirculatory parameters indicates that the onset of MOF is mainly influenced by microvascular factors, and the correlation between microcirculatory variables and mtOM damage without the involvement of transient loss of oxidative capacities further confirms the presence of strong mitochondrial coping mechanisms against membrane damage.

Mitochondrial dysfunction can occur in sepsis both with and without shock as well, but it is not uniformly present. The pathogenesis is complex and still only partially known, but it seems that different mitochondrial functionalities may be present in adjacent areas with microvascular flow heterogeneity^36^. The non-uniformity may also result from the heterogeneous ultrastructural, biophysical and electrochemical characteristics of liver mitochondria, which may lead to potentially decaying and surviving subpopulations in response to pathologies and post-translational processes^37^. Heterogeneity may also arise from the mitochondrial life cycle itself, including biogenesis, motility, fusion and fission, and the clearance of damaged mitochondria. In this line, damaged mitochondria can fuse with other mitochondria with intact membranes, and a regenerative fusion-fission phenomenon was already observed during sepsis progression. Thus, we hypothesize that increased CytC efflux preceded the complete clearance of degraded mitochondria with outer membrane damage and that a structurally and metabolically more resilient and compensatory subpopulation was able to maintain undisturbed ATP synthesis. These mitochondria may be maintainers or restorers of microcirculation and tissue integrity in the later period where sepsis is resolved^38^.

In this study design, the ROFA score was used to monitor MOF progression similarly to human clinical routine, and the diagnostic value of microcirculatory parameters could be established within this scheme. As with human practice, the serum lactate level was also significantly elevated and proved to be a strong predictor of severity, supporting the diagnostic importance of hypoxia markers^39^. However, the low ALT, AST and urea levels all point to the direction of “septic stunning”, suggesting that the organs were primed with “hypoxic hibernation” for the expected insult.

Braeley et al. have described marked changes in mitochondrial functions^8,9^, but microcirculation and MOF have not yet been detected simultaneously. We aimed to complement these measurements by using the latest consensus recommendations, a standardized MOF severity score system and simultaneous microcirculation measurements. Our experimental protocol provided an opportunity to address the chicken or the egg causality dilemma of MMDS in sepsis. Microcirculation is a significantly earlier predictor of multi-organ failure as compared to mitochondrial respiration. We have used particular parameters to demonstrate that detectable microcirculatory or mitochondrial functional deteriorations occur at different time points depending on the course of sepsis and the compensatory mechanisms and that inappropriate monitoring times may lead to erroneous conclusions. By sequentially tracking the development of MOF, our results shed light on the dynamics of the microvascular oxygen supply, thus providing a better chance for the appropriate timing of diagnostic and therapeutic efforts.

The results demonstrate that an early, marked microcirculatory dysfunction was followed during this time by late and presumably compensatory mitochondrial changes. Our study has certain limitations, which may warrant further investigation. The points of the latest MQTiPSS recommendations were followed, but antibiotics were omitted from the protocol due to their incompletely mapped mitochondrial side effects^40^. In terms of progression, it would have been preferable to allow a longer follow-up. Given the approx. 1:21 ratio of rat time–human time conversion^41^, we can assume that the acute phase was fully covered, but additional studies are needed to explore the later effects in later phases of sepsis. Due to the dominant portal blood supply of the liver, the incoming hypoxic signals from the intestines may influence the hepatic mitochondrial functions. Based on this consideration parallel microcirculation and mitochondrial study of the same organ would have been the perfect solution to test our hypothesis, but technical limitations precluded this possibility (intravital videomicroscopy of the liver is influenced by the unique circulation of the sinusoidal structure and the darkness of the tissue itself^42^. Meanwhile, evaluation of intestinal mitochondrial functions is hindered by the high linolenic and oleic acid content, as these molecules can damage the mitochondrial membranes^43^. A future experiment should aim the structural and functional separation of the putative mitochondrial subpopulations could best be investigated in isolated in vitro systems, which did not fit into the present experimental set-up.

We designed a rat model of intraabdominal sepsis, and we described the splanchnic microcirculation- and mitochondrial respiration-linked changes in detail with simultaneous tracking. Microvascular perfusion insufficiency proved to be the route of progression and an early predictor of the initiation of MOF. Further, the later decline and rapid recovery of oxidative mitochondrial functions suggest the existence of mitochondrial subpopulations with different responses to tissue hypoxia.

## Methods

### Animals

Male Sprague–Dawley rats (350 ± 30 g) were housed in plastic cages in a temperature-controlled (21–23 °C) room with a daily 12/12-h light and dark cycle and access to standard rodent food and water ad libitum. Procedures were performed in accordance with NIH guidelines and EU directive 2010/63/EU for the protection of animals used for scientific purposes, and the study was approved by the National Scientific and Ethical Committee, the national competent authority of Hungary. (ETT-TUKEB; license number: V/175/2018).

### Sepsis induction and experimental protocol

Sample size was estimated assuming approx. 20% mortality after 24 h. If the presumed true hazard ratio of septic subjects relative to controls is 0.2 with a power of 1 − β = 0.8 and the Type I error probability is α = 0.05, the inclusion of 8 septic and 8 control animals was recommended for each time point that had been selected. In line with the sample size estimation, the animals were randomly assigned to sham-operated (n_∑_ = 40) and septic groups (n_∑_ = 40), which were randomly further divided into five independent subgroups each (n_12h_ = 8, n_16h_ = 8, n_20h_ = 8, n_24h_ = 8, n_28h_ = 8) according to the different time of sepsis progression.

Figure 1a provides a depiction of the experimental protocol. Sepsis was induced with intraperitoneally (ip) administered fecal inoculum, while fresh feces (4 g) samples were collected, filtered and suspended in physiological saline as described previously^21,44^. Sham-operated animals received the same volume of sterile saline ip. After 6, 12, 16, 20 or 24 h of sepsis progression, the animals received fluid therapy (Ringerfundin, 1.5 mL/kg subcutaneously; B. Braun, Melsungen, Germany) and analgesics (buprenorphine, 15 μg/kg subcutaneously; Richter Pharma, Hungary), and assessments of animal well-being (RSS) was performed. The animals were anesthetized with a mixture of ketamine (45.7 mg/kg) and xylazine (9.12 mg/kg) ip, and invasive hemodynamic monitoring was started after a 30-min stabilization period. The study design and the presentation of the data are in accordance with the Minimum Quality Threshold in Pre-Clinical Sepsis Studies (MQTiPSS) recommendations and considerations^45^ and with the Animal Research: Reporting of In Vivo Experiments guidelines (https://arriveguidelines.org/).

### Surgical preparation and invasive measurements

The anesthetized animals were placed on a heating pad (37 °C) in a supine position. A tracheostomy was performed to support and provide spontaneous breathing. The right external jugular vein was cannulated for fluid replacement (Ringerfundin, 10 mL/kg/h) and for the maintenance of continuous anesthesia (ketamine 12 mg/kg/h, xylazine 2.4 mg/kg/h and diazepam 0.576 mg/kg/h iv.) during the 60-min monitoring period. The left common carotid artery was cannulated to monitor mean arterial pressure (MAP) and a thermistor-tip catheter was positioned into the contralateral common carotid artery to measure cardiac output (CO) using a thermodilution technique (SPEL Advanced Cardiosys 1.4, Experimetria Ltd., Budapest, Hungary). CO was indexed for body weight. Hemodynamic parameters were measured every 15 min for 60 min. Arterial blood samples were taken for blood gas analyses (Cobas b123; Roche Ltd., Basel, Switzerland) at the 60th min of the monitoring period. The global oxygen delivery (DO_2_ = CO × [(1.38 × Hb × SaO_2_) + (0.003 × PaO_2_)]), oxygen consumption (VO_2_ = CO × [(1.38 × Hb × (SaO_2_ − SvO_2_)) + (0.003 × PaO_2_)]) and oxygen extraction (ExO_2_ = DO_2_/VO_2_) values were calculated from these parameters. The lung function was determined by using the arterial partial pressure of oxygen to fraction of inspired oxygen (PaO_2_/FiO_2_, where FiO_2_: 0.21) ratio. After the 60-min hemodynamic monitoring period, a median laparotomy was performed to observe the microcirculation of the ileal serosa. A liver tissue biopsy was taken immediately after microcirculatory measurements to evaluate mitochondrial respiratory functions from homogenate. After tissue samplings, animals were sacrificed under deep anesthesia with an overdose of ketamine (120 mg/kg).

### Measurements of metabolic, inflammatory and organ dysfunction markers

Whole blood lactate levels were measured from venous blood samples (Accutrend Plus Kit; Roche Diagnostics Ltd., Rotkreuz, Switzerland). Blood samples were collected from the inferior vena cava into EDTA-coated tubes (1 mg/mL), centrifuged (1.200*g* at 4 °C for 10 min) and stored at − 70 °C. Plasma interleukin-6 (IL-6) level was determined according to the standard ELISA kit protocol (Cusabio Biotechnology Ltd., Wuhan, China). Kidney function was characterized by plasma urea level. Liver function was assessed by measuring plasma alanine aminotransferase (ALT) and aspartate aminotransferase (AST) levels using a Roche/Hitachi 917 analyzer (F. Hoffmann-La Roche AG, Switzerland). All analyses were performed in a blinded experimental set-up on coded samples.

### Rat organ failure assessment (ROFA) scores

In accordance with the considerations of the Sepsis-3 and MQTiPSS consensus guidelines, the ROFA^21,46^ scoring system was used to describe the severity of multiorgan failure. The scoring system considers the cardiovascular (MAP), respiratory (PaO_2_/FiO_2_ ratio), hepatic (ratio of AST/ALT or De Ritis ratio), renal (urea level) and global metabolic (lactate level) dysfunctions. Sepsis was defined as a cumulative ROFA score over 2.

### Microcirculatory measurements

The microcirculation of the ileal serosa was visualized with the Incident Dark Field (IDF) imaging technique (CytoCam Video Microscope System; Braedius Medical, Huizen, the Netherlands) as described earlier^44,46^. The device consists of special LEDs emitting guided light with a wavelength of 530 nm, absorbed by hemoglobin-containing particles, thus the vascular bed appears as a “network of black dots on a grey background”. Thanks to the real-time tracking IDF differentiates the rate of blood flow from the movement of the red blood cells^47^. Images from an ileum segment were recorded in six, 50-frame, high-quality video clips. The records were analyzed with an offline software-assisted system (AVA 3.0, Automated Vascular Analysis, Academic Medical Center, University of Amsterdam). The proportion of perfused vessels (PPV) was defined as the ratio of the perfused vessel lengths to total vessel lengths: (total number of vessels—[no flow + intermittent flow]/total number of vessels) × 100^48^. Microvascular flow index (MFI) was randomly and blindly determined by a single researcher. After a semiquantitative analysis by eye, individual vessels were distinguished between no flow (0), intermittent flow (1), sluggish flow (2), and continuous flow (3). A value was assigned to each vessel and the overall score of each record was the average of the individual values. The heterogeneity index (HI) was defined as the difference between the highest MFI and the lowest MFI divided by the average MFI of the record: (MFI_highest_ − MFI_lowest_)/MFI_average_^46,49,50^. The equation used in calculating the microvascular parameters is shown in Supplementary Equation S1.

### Assessment of liver mitochondrial function

Mitochondrial VO_2_ (mtVO_2_) was measured in liver homogenates using High-Resolution FluoRespirometry (Oxygraph-2 k, Oroboros Instruments, Innsbruck, Austria) as described earlier^44^. In brief, liver samples obtained from the left lateral lobe were homogenized with a tissue grinder Potter–Elvehjem, and then respirometry was performed in a MiR05 respiration medium at constant temperature and with continuous stirring (37 °C, 750 rpm). After stabilization of O_2_ consumption, rotenone was added (1) to inhibit complex I activity and (2) to prevent accumulation of oxaloacetate (a known endogenous inhibitor of complex II). FADH_2_-supported leak respiration and maximal capacity of oxidative phosphorylation (OxPhos) were determined in the presence of succinate and adenosine diphosphate (ADP). Following stimulation of OxPhos, the integrity of the mitochondrial outer membrane (mtOM) was tested with the addition of exogenous cytochrome c, and the increase in mtVO_2_ was expressed as a percentage of mtVO_2_ as compared to mtVO_2_ in OxPhos (CytC%). Complex V (or ATP synthase) was inhibited by oligomycin to assess leak respiration in a non-phosphorylating state (LEAK_Omy_) and to calculate respiratory control ratio (RCR), an index of the coupling of mitochondrial respiration to OxPhos (Oxphos/LEAK_Omy_). DatLab 7.3.0.3. Software (Oroboros Instruments, Innsbruck, Austria) was used for online display, respirometry data acquisition and analysis.

### Statistical analysis

Data were evaluated with the SigmaStat 13 software package (Systat Software, San Jose, CA). Survival rate was analyzed and plotted using the Kaplan–Meier method. The Mann–Whitney or Kruskal–Wallis tests were used with Dunn’s post-hoc test for discrete (score) variables, while a two-way analysis of variance was used for continuous variables followed by the Holm–Sidak post-hoc test. Data were displayed as median values and interquartile ranges between the 75th and 25th percentiles, with P < 0.05 being considered significant. The receiver operating characteristic (ROC) curve analysis was used to evaluate the classification power to represent the diagnostic accuracy of the different parameters. A multivariate analysis model was performed with a logical data analysis method and data processing with GraphPad Prism 8.0 software. Pearson’s method (for the ROFA score) or Spearman’s methods were used to analyze linear correlation; correlation coefficient (r), regression lines and 95% confidence intervals were indicated.

### Supplementary Information

Supplementary Information.

## Data Availability

The datasets generated during and/or analyzed during the current study are available from the corresponding author on reasonable request.

## References

[1] Singer M (2016). The third international consensus definitions for sepsis and septic shock (sepsis-3). J. Am. Med. Assoc..

[2] Ince C (2005). The microcirculation is the motor of sepsis. Crit. Care..

[3] Mongardon N, Dyson A, Singer M (2009). Is MOF an outcome parameter or a transient, adaptive state in critical illness?. Curr. Opin. Crit. Care..

[4] De Backer D (2013). Microcirculatory alterations in patients with severe sepsis: Impact of time of assessment and relationship with outcome. Crit. Care. Med..

[5] Moore JPR, Dyson A, Singer M, Fraser J (2015). Microcirculatory dysfunction and resuscitation: Why, when, and how. Br. J. Anaesth..

[6] Balestra GM, Legrand M, Ince C (2009). Microcirculation and mitochondria in sepsis: Getting out of breath. Curr. Opin. Anaesthesiol..

[7] Jarczak D, Kluge S, Nierhaus A (2021). Sepsis-pathophysiology and therapeutic concepts. Front. Med. Lausanne..

[8] Brealey D (2004). Mitochondrial dysfunction in a long-term rodent model of sepsis and organ failure. Am. J. Physiol. Regul. Integr. Comp. Physiol..

[9] Mesquida J (2012). Prognostic implications of tissue oxygen saturation in human septic shock. Intensive Care Med..

[10] Sakr Y, Dubois MJ, De Backer D, Creteur J, Vincent JL (2004). Persistent microcirculatory alterations are associated with organ failure and death in patients with septic shock. Crit. Care Med..

[11] Spronk, P.E. Microcirculatory and Mitochondrial Distress Syndrome (MMDS): A New Look at Sepsis. in *Functional Hemodynamic Monitoring: Update in Intensive Care and Emergency Medicine.* (ed. Pinsky, M.R., Payen, D.) 47–67 (Springer, 2005).

[12] Clowes GH, O’Donelli TF, Ryan NT (1974). Energy metabolism in sepsis: Treatment based on different patterns in shock and high output stage. Ann. Surg.

[13] Lang CH, Bagby GJ, Ferguson JL, Spritzer JJ (1984). Cardiac output and redistribution of organ blood flow in hypermetabolic sepsis. Am. J. Physiol..

[14] Protti A, Singer M (2006). Bench-to-bedside review: Potential strategies to protect or reverse mitochondrial dysfunction in sepsis-induced organ failure. Crit. Care..

[15] Singer M, De Santis V, Vitale D, Jeffcoat W (2004). Multiorgan failure is an adaptive, endocrine-mediated, metabolic response to overwhelming systemic inflammation. Lancet..

[16] Standage SW (2017). PPARα augments heart function and cardiac fatty acid oxidation in early experimental polymicrobial sepsis. Am. J. Physiol. Heart. Circ. Physiol..

[17] Preau S (2021). Energetic dysfunction in sepsis: A narrative review. Ann. Intensive. Care..

[18] Brealey D (2002). Association between mitochondrial dysfunction and severity and outcome of septic shock. Lancet..

[19] Kohoutová M, Dejmek J, Tůma Z (2018). Variability of mitochondrial respiration in relation to sepsis-induced multiple organ dysfunction. Physiol. Res..

[20] Donati A (2013). From macrohemodynamic to the microcirculation. Crit. Care. Res. Pract..

[21] Tallósy SP (2021). The microbial composition of the initial insult can predict the prognosis of experimental sepsis. Sci. Rep..

[22] Bauer M (2020). Mortality in sepsis and septic shock in Europe, North America and Australia between 2009 and 2019—Results from a systematic review and meta-analysis. Crit. Care..

[23] Ellis CG, Bateman RM, Sharpe MD, Sibbald WJ, Gill R (2002). Effect of a maldistribution of microvascular blood flow on capillary O(2) extraction in sepsis. Am. J. Physiol. Heart Circ. Physiol..

[24] Kowalewska PM (2022). Spectroscopy detects skeletal muscle microvascular dysfunction during onset of sepsis in a rat fecal peritonitis model. Sci. Rep..

[25] Clark JA, Coopersmith CM (2007). Intestinal crosstalk: A new paradigm for understanding the gut as the "motor" of critical illness. Shock..

[26] Nakajima Y, Baudry N, Duranteau J, Vicaut E (2001). Microcirculation in intestinal villi: A comparison between hemorrhagic and endotoxin shock. Am. J. Respir. Crit. Care. Med..

[27] Mammen EF (1998). The haematological manifestations of sepsis. J. Antimicrob. Chemother..

[28] Iba T, Levy JH (2020). Sepsis-induced coagulopathy and disseminated intravascular coagulation. Anesthesiology..

[29] Spronk PE, Zandstra DF, Ince C (2004). Bench-to-bedside review: Sepsis is a disease of the microcirculation. Crit. Care..

[30] Sullivan RC, Rockstrom MD, Schmidt EP, Hippensteel JA (2021). Endothelial glycocalyx degradation during sepsis: Causes and consequences. Matrix Biol. Plus..

[31] Borutaite V, Budriunaite A, Morkuniene R, Brown GC (2001). Release of mitochondrial cytochrome c and activation of cytosolic caspases induced by myocardial ischaemia. Biochim. Biophys. Acta..

[32] Andersen LW (2016). Cytochrome C in patients with septic shock. Shock..

[33] Adachi N (2004). Serum cytochrome c level as a prognostic indicator in patients with systemic inflammatory response syndrome. Clin. Chim. Acta..

[34] Kay L, Daneshrad Z, Saks VA (1997). Alteration in the control of mitochondrial respiration by outer mitochondrial membrane and creatine during heart preservation. Cardiovasc. Res..

[35] Eleftheriadis T, Pissas G, Liakopoulos V, Stefanidis I (2006). Cytochrome c as a potentially clinical useful marker of mitochondrial and cellular damage. Front. Immunol..

[36] Ince C, Mik EG (2016). Microcirculatory and mitochondrial hypoxia in sepsis, shock, and resuscitation. J. Appl. Physiol..

[37] Ngo J, Osto C, Villalobos F, Shirihai OS (2021). Mitochondrial heterogeneity in metabolic diseases. Biology (Basel)..

[38] Chen H, Chomyn A, Chan DC (2005). Disruption of fusion results in mitochondrial heterogeneity and dysfunction. J. Biol. Chem..

[39] Zhai X (2018). Lactate as a potential biomarker of sepsis in a rat cecal ligation and puncture model. Mediators Inflamm..

[40] Suárez-Rivero JM (2021). Mitochondria and antibiotics: For good or for evil?. Biomolecules..

[41] Sengupta P (2013). The laboratory rat: Relating its age with human's. Int. J. Prev. Med..

[42] Uz Z (2020). Intraoperative imaging techniques to visualize hepatic (micro)perfusion: An overview. Eur. Surg. Res..

[43] Stanbury PJ (1961). Comparison of the mitochondria of the small intestine of vertebrates. Nature..

[44] Rutai A (2019). Endothelin A and B receptors: Potential targets for microcirculatory-mitochondrial therapy in experimental sepsis. Shock..

[45] Osuchowski MF (2018). Minimum quality threshold in pre-clinical sepsis studies (MQTiPSS): An international expert consensus initiative for improvement of animal modeling in sepsis. Shock..

[46] Juhász L (2020). Divergent effects of the N-methyl-D-aspartate receptor antagonist kynurenic acid and the synthetic analog SZR-72 on microcirculatory and mitochondrial dysfunction in experimental sepsis. Front. Med. Lausanne..

[47] Aykut G, Veenstra G, Scorcella C, Ince C, Boerma C (2015). Cytocam-IDF (incident dark field illumination) imaging for bedside monitoring of the microcirculation. Intensive Care Med Exp..

[48] De Backer D, Creteur J, Preiser JC, Dubois MJ, Vincent JL (2002). Microvascular blood flow is altered in patients with sepsis. Am. J. Respir. Crit. Care Med..

[49] Ince C (2018). Second consensus on the assessment of sublingual microcirculation in critically ill patients: Results from a task force of the European Society of Intensive Care Medicine. Intensive Care Med..

[50] Pozo MO, Kanoore Edul VS, Ince C, Dubin A (2012). Comparison of different methods for the calculation of the microvascular flow index. Crit. Care Res. Pract..

