# Supplementary material for: Microcirculation-driven mitochondrion dysfunction during the progression of experimental sepsis
Source: Sci Rep. 2024 Mar 26;14:7153. doi: 10.1038/s41598-024-57855-9 (PMC10966066; doi:10.1038/s41598-024-57855-9)
Supplement: Supplementary file 1 — Supplementary Information. [file 41598_2024_57855_MOESM1_ESM.docx]

**Supplementary Material**

**Microcirculation-driven Mitochondrion Dysfunction during the Progression of Experimental Sepsis**

Roland Fejes, Attila Rutai, László Juhász, Marietta Zita Poles, Andrea Szabó, József Kaszaki, Mihály Boros, Szabolcs Péter Tallósy*

Institute of Surgical Research, Albert Szent-Györgyi Medical School, University of Szeged

***e-mail:** [tallosy.szabolcs@med.u-szeged.hu](mailto:tallosy.szabolcs@med.u-szeged.hu)


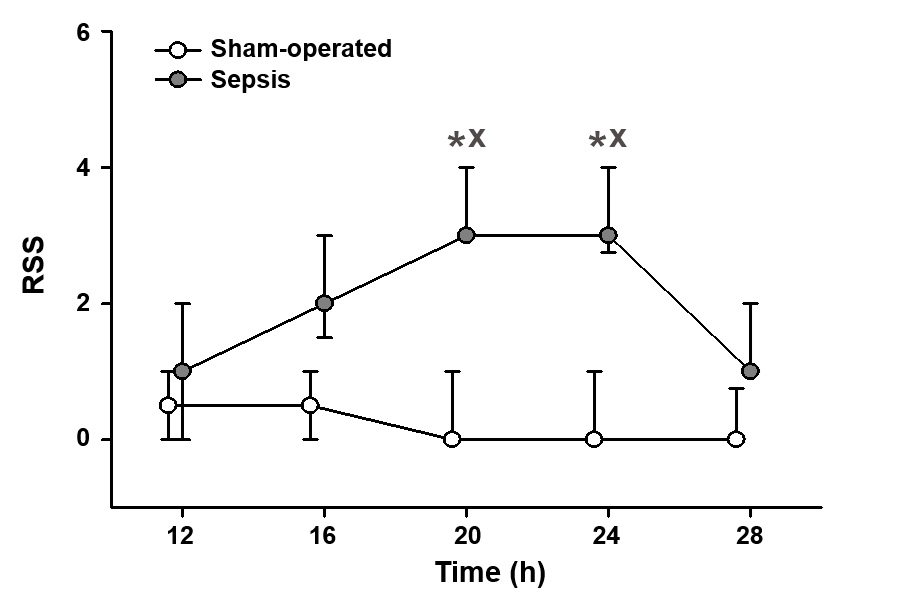


**Supplementary Figure S1.** Well-being-related sickness score (RSS) shows the general suffering of animals due to sepsis induction. A comparison within groups was conducted with the Kruskal-Wallis test followed by Dunn’s post-hoc test, compared to the 12^th^ hr group. The Mann-Whitney U test was performed between sham-operated and septic groups at the same time point. **P* < 0.05 vs. sham-operated within the same time point, ^X^*P* < 0.05 vs. 12^th^ hr group within treatment.

**Supplementary Figure S2.** Time dependent fold changes of major variables of the study. Fold changes of ROFA score, mean arterial pressure (MAP), cardiac output (CO), proportion of perfused vessels (PPV), heterogeneity index (HI), oxidative phosphorylation (OxPhos) and cytochrome c control efficiency (CytC%) in the septic animals can be seen.

$$\boldsymbol{PPV}\mathbf{=}\frac{Length of perfused vessels}{Length of all recorded vessels} x 100$$

$$\boldsymbol{HI}\mathbf{=}\frac{highest MFI value-lowest MFI value}{average MFI value}$$

**Supplementary Equation S1.** Equations used for the numerical representation of major microcirculatory features. PPV means proportion of perfused vessels, HI means heterogeneity index, while MFI means microvascular flow index.
